# Improving the anti-fouling property and permeate flux of hollow fiber composite nanofiltration membrane using β-cyclodextrin

**DOI:** 10.1038/s41598-019-48908-5

**Published:** 2019-08-27

**Authors:** Yuantao He, Jing Miao, Zhibin Jiang, Kai Tu, Hao Yang, Shunquan Chen, Ling Zhang, Rui Zhang

**Affiliations:** 1 Guangdong Key Laboratory of Membrane Materials and Membrane Separation, Guangzhou Institute of Advanced Technology, Chinese Academy of Sciences, Nansha District, Guangzhou, 511458 China; 2 Key Laboratory for Green Chemical Process of Ministry of Education, School of Environmental Ecology and Biological Engineering, Wuhan Institute of Technology, Wuhan, 430205 China; 3 School of Resource and Environment, University of Jinan, Jinan, 250022 China; 4 R & D Center, Sinochem Ningbo River Membrane Technology Corp. Ltd., Beijing, China; 5 Shenzhen Institute of Advanced Technology, Chinese Academy of Sciences, Shenzhen, 518055 China

**Keywords:** Environmental chemistry, Pollution remediation

## Abstract

Hollow fiber composite NF membranes with improved anti-fouling property and water flux were prepared via interfacial polymerizationand layer-by-layer method using polyethylenimine (PEI), isophthaloyl dichloride (IPC), and β-cyclodextrin (β-CD). The chemical structures and the morphologies of the resultant NF membranes were characterized by attenuated total reflectance-fourier transform infrared (ATR-FTIR) spectroscopy and scanning electron microscopy (SEM). The effects of β-CD concentration on membrane morphologies, nanofiltration performances, surface hydrophilicities and anti-fouling properties were investigated. It was found that the permeate flux increased with increasing the β-CD concentration, and no decline of rejection was observed. The results showed that the introduction of β-CD improved surface hydrophilicities and anti-fouling performances of composite hollow fiber NF membranes. The water contact angles decreased from 61.3° to 23° within creasing the concentration of β-CD from 0 to 2.0 wt.%. The resultant hollow fiber composite NF membrane showed an excellent anti-fouling property with the flux recovery ratio of 97.6%, which was much better than that of the original polyamide (PA) NF membranes.

## Introduction

With the development of the society, the shortage of water sources and the environmental pollution led to an urgent demand for production and recycling of water sources^1–3^. As a novel and promising technology, membrane technology is playing an important role in water treatment because of its high efficiency and low cost^4,5^. Nanofiltration (NF) membrane is a kind of pressure driven membrane with separation properties between reverse osmosis (RO) and ultrafiltration (UF) membranes^6–8^. Due to the advantages of low operating pressure, high permeate flux, and iron perm-selectivity. NF membranes have been widely applied to various industrial fields^9,10^, such as seawater desalination^11^, industrial wastewater treatment^12^, purification of drinking water^13^, biotechnology^14^, and food science^15^.

Polyamide (PA) NF membrane is the most common NF membrane in the market. Commercial NF membranes are mostly obtained via interfacial polymerization of binary amines or polyamine and acyl chloride on the surface of UF membranes^16,17^. However, the polyamide active layer would be fouled easily by microorganisms, colloids, inorganic scaling, and organic compounds^18–20^. The membrane pores will be blocked up by those pollutants^21^. As a result, various negative effects including flux decline, increase of operating pressure, increment of energy consumption, and membrane degradation^22,23^. Several strategies have been applied to improve the anti-fouling properties of NF membranes^24,25^. Most methods could be summarized as the hydrophilic modification and the incorporation of hydrophilic components. An, *et al*.^26^ developed an anti-fouling NF membrane by adding different amounts of polyvinyl alcohol (PVA) into piperazine (PIP) during its interfacial polymerization with trimesoyl chloride (TMC). The hydrophilicity and the permeate flux of the composite NF membranes increased with increasing the mass fraction of PVA to PIP, and the anti-fouling property was improved significantly. Zhang, *et al*.^27^ have prepared NF membranes by introducing sulfonated multiwall carbon nanotube (SMWCNT) to the poly (piperazine amide) thin-film nanocomposite (TFN) membranes. The water contact angle (WCA) gradually decreased with increasing the concentration and the pure water permeability (*PWP*) remarkably increased which also exhibited better anti-fouling ability to BSA. Mi, *et al*.^28^ grafted zwitterionic component (N-aminoethyl piperazine propane sulfonate, AEPPS) onto polyamide thin film composite NF membranes that were prepared via interfacial polymerization between TMC and PIP. The hydrophilicity and the permeation flux of the composite NF membranes were higher than the pristine polyamide membrane. The zwitterionic NF membranes also exhibited good anti-fouling properties.

Cyclodextrin (CD) are cyclic oligosaccharides composed of several glucose units linked by α-(1–4) bonds with a tortuous-shaped structure^29^. In the tortuous-shaped structure, the upper outer end (larger open end) is composed of secondary hydroxyl groups of C_2_ and C_3_, and the lower end (smaller open end) is composed of primary hydroxyl groups of C_6_, which are hydrophilic^30,31^, and the hydrophobic zone is formed in the cavity due to the shielding effect of C-H bond^32^. With the special structure characterized by a hydrophilic external surface and a relative hydrophobic cavity, a channel is formed that make it easier for water to pass through^33,34^. Due to numerous hydrophilicity groups and the special structure of CDs, it is expected to improve the permeate flux and the anti-fouling properties of NF membranes^35,36^. Besides, β-CD is composed of 6 glucose units, of low-priced and generally the most used CDs. Wu, *et al*.^37^ fabricated a polyester thin-film composite NF membrane via interfacial polymerization of TMC and triethanolamine (TEOA) in the presence of β-CD. The prepared NF membrane showed an enhancement in the anti-fouling performance. However, the NF membrane showed a decline in the rejection with the increase of the β-CD concentration.

**Figure 1:**
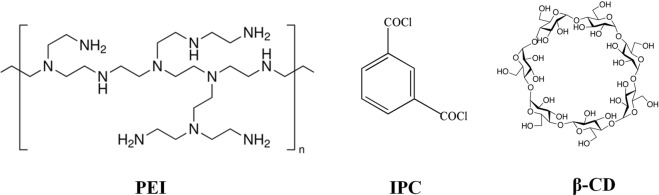
Molecularstructures of PEI, IPC and β-CD molecules.

In this work, hollow fiber composite NF membranes with improved anti-fouling property and water flux were prepared via interfacial polymerization and layer-by-layer method using polyethylenimine (PEI), isophthaloyl dichloride (IPC) and β-CD. Figure 1 shows the molecular structures of PEI, IPC, and β-CD. For the first time, the β-CD was used to modify the polyamide composite NF membrane. Firstly, a polyamide layer was prepared by the IP between PEI and IPC on the surface of Polyvinylidene fluoride (PVDF) UF membrane. Then the residual acyl chloride reacted with β-CD monomers after immersing the polyamide NF in the aqueous solution containing β-CD monomers. Finally, a polyester structure was formed, and the surfaces of PA NF membrane were modified by hydrophilic β-CD monomers. The effects of β-CD on the rejection performances, the morphology, the hydrophilicity, the chemical structure, and the anti-fouling performance of the composite NF membrane were investigated.

## Materials and Methods

### Materials

Polyvinylidene fluoride (PVDF) hollow fiber ultrafiltration (UF) membrane with MWCO of 30 K Da was purchased from Hangzhou Yuanxiang Film Co. Ltd (Hangzhou, China). The outer diameter of PVDF hollow fiber UF membrane is 1.4 mm. The pure water permeation (*PWP*) of PVDF hollow fiber UF membrane is 62 L·m^−2^·h^−1^·bar. PEI was purchased from Gobekie New Material Technology Co. Ltd (Shanghai, China). β-cyclodextrin (β-CD, 98%) and isophthaloyl dichloride (IPC, 98%) was obtained from Shanghai Macklin Biochemical Co. Ltd (Shanghai, China). n-hexane was purchased from Kermel Chemical Agent Co. Ltd (Tianjin, China). Bovine serum albumin (BSA, 99%) was purchased from Shanghai Rebiosci Biotechnology Co. Ltd (Shanghai, China). Inorganic salts, including magnesium chloride (MgCl_2_), sodium sulfate (Na_2_SO_4_), magnesium sulfate (MgSO_4_), were obtained from Sinopharm Chemical Reagent Co. Ltd (Shanghai, China). All reagents were of analytical grade (AR) without further purification.

### Preparation of PEI/IPC/CD hollow fiber composite NF membranes

**Figure 2:**
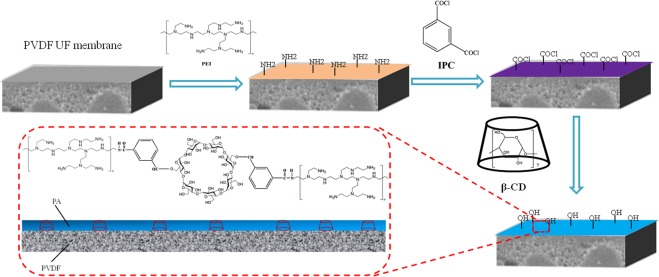
Schematic diagram of preparation of PEI/IPC/β-CD hollow fiber composite NF membrane.

After being pre-soaked for 24 h in deionized (DI) water for removing contaminants, the PVDF UF membranes were immersed in the 4.0 wt.% PEI aqueous solution. After being soaked for 5 min, the excess PEI aqueous solution was rinsed off from the membrane surface. Next, these membranes were immersed in 0.25 wt.% IPC in n-hexane for 90 s. After removing the excess IPC solution, the membranes were immersed in the β-CD aqueous solution immediately for 30 min, and then cured for 30 min in an oven to stabilize the structure. The hollow fiber composite membranes were prepared with β-CD concentration at 0, 0.5, 1.0, 1.5, 2.0 wt.% were labeled as NF-0, NF-1, NF-2, NF-3, and NF-4, respectively. Schematic diagram of preparation of PEI/IPC/β-CD hollow fiber composite NF membrane was showed in Fig. 2. Finally, the resultant hollow fiber composite PEI/IPC/CDNF membranes were sealed in a polymethyl methacrylate (PMMA) pipe to obtain the hollow-fiber membrane modules. The outer diameter and the inner diameter of PMMA pipe were 15 mm and 11 mm, respectively. The hollow fiber composite NF membrane was placed in the PMMA pipe, both ends of the tube and the membrane were sealed with epoxy adhesive. After the adhesive was solidified, another end of the pipe was also sealed, while another end of the hollow fiber composite NF membrane should be unobstructed. Two holes were drilled on the pipe for the circulation of the solution.

### Characterizations of hollow fiber composite NF membranes

The surface and the cross-section morphologies of PVDF UF membranes and the as-prepared hollow fiber composite NF membranes were characterized using a scanning electron microscope (SEM, Phenom ProX, Netherlands). The attenuated total reflectance-Fourier transform infrared (ATR-FTIR) spectra were collected in the range of 700 to 4000 cm^−1^ at a resolution of 4 cm^−1^ with a bench-top IR spectrometer (FTIR920, Tianjin Tuopu, China), to characterize the chemical structures of the resultant composite NF membrane surfaces. The hydrophilicities of the membrane surfaces were characterized with the static contact angle measurements (DSA305, Kruss GmbH, Germany) using 0.2 μL droplet of pure water.

Rejection performance including permeate flux (*F*) and rejection rate (*R*) were measured with a lab-scale cross-flow membrane evaluation apparatus at 25 °C. The texts were carried out with 1000 ppm Na_2_SO_4_, MgSO_4_, and MgCl_2_ aqueous solutions at an operating pressure of 0.2 MPa. Before test, the system was first pressurized at 0.3 MPa for 1 h. The permeation flux (*F*) was calculated using the following equation.

1 $${\rm{F}}={\rm{V}}/({\rm{S}}\cdot {\rm{t}})$$

where, *V* is the volume of permeated aqueous solution during the measurement (m^3^), *S* is the effective membrane area (m^2^), *t* is the time period for measurement (h).

The rejection rate (*R*) was calculated using the following equation:

2 $${\rm{R}}=100\times (1-({{\rm{C}}}_{{\rm{p}}}/{{\rm{C}}}_{{\rm{f}}}))$$

Where, *C*_*p*_ and *C*_*f*_ (mol/L) are the salt concentration in the permeate and the feed solution. The salt concentrations were obtained with the standard curves of different salts, which show the corresponding relations between the salt concentrations and the electrical conductivities of the salt aqueous solutions. The electrical conductivities of the salt aqueous solutions were measured with a DDS-11A conductivity meter (Shanghai Leici Instrument Company, China). All experimental results were the average values of 5 measurements.

### Anti-fouling performance test

The anti-fouling properties of NF membranes were tested with a solution of bovine serum albumin (BSA) at 0.2 MPa and room temperature. DI water was firstly filtered through NF membrane for at least 1 h and the average water flux was recorded as *J*_0_. The water flux was recorded every 20 min in the continuous 80-min running. Then the BSA solution (1 g/L) was filtered through the membrane, and the average water flux was recorded as *J*_*f*_. The permeate flux was measured every 20 min in the continuous 80 min running as well. The membrane was washed with DI water for 30 min, and then the operation was repeated once. The antifouling performance evaluation for each membrane was operated with 2.5 cycles. Normalized flux (*J*_*f*_ /*J*_0_) is used to evaluate the anti-fouling performance of NF membranes.

## Result and Discussion

### Chemical structures of membranes

**Figure 3:**
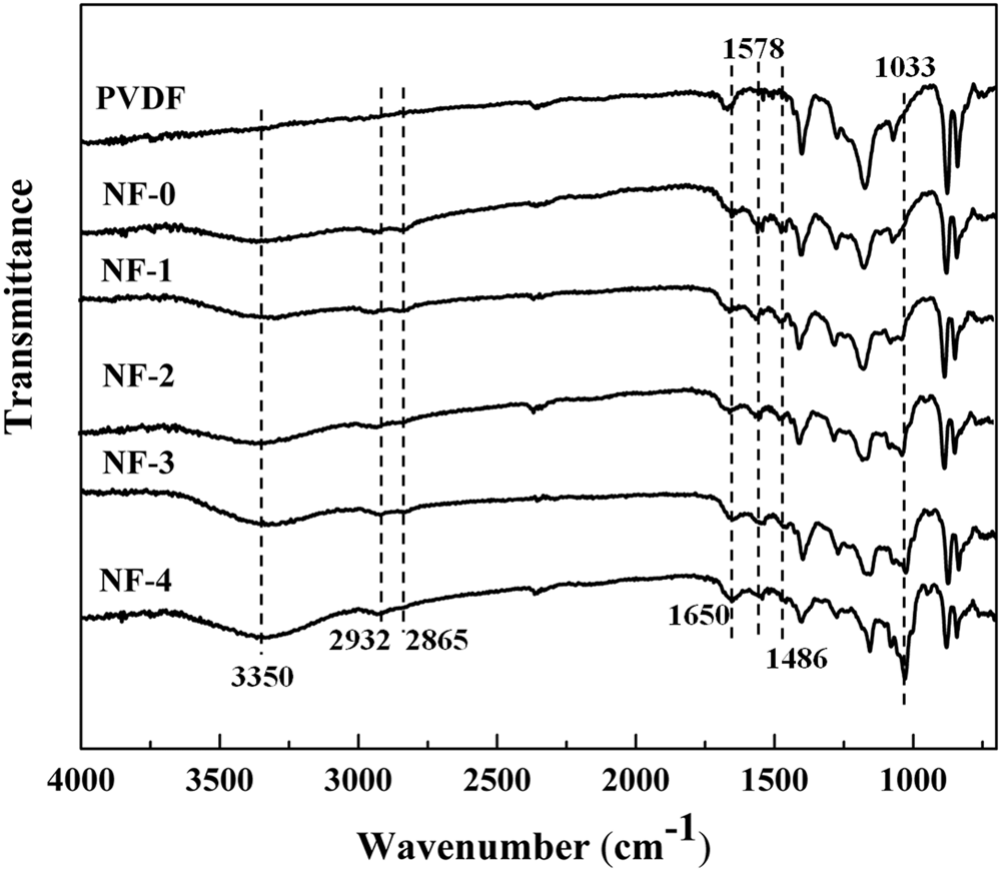
ATR-FTIR spectra of PVDF UF substrate and different resultant composite NF membranes.

ATR-IR spectra were utilized to analyze the chemical structures of PVDF UF membrane and NF membranes, as shown in Fig. 3. Comparing with PVDF membrane, the new absorbance peaks occurred at 3350 cm^−1^, which is attributed to the overlap of the amine groups (-N-H) and the hydroxyl groups (-O-H) stretching vibrations, which could be associated with the excess of β-CD reactants. Besides, the absorbance peaks were enhanced with the increase of β-CD concentration. The absorbance peaks at 2932 and 2865 cm^−1^ are characteristic of the C–H stretching vibrations of a saturated aliphatic series. The new absorbance peaks presented at 1650 cm^−1^ could be assigned to the carbonyl group (–C=O) of the amide groups. The bands at 1578 cm^−1^ and 1486 cm^−1^ correspond to the stretching vibration of C=C skeleton in aromatic ring. Moreover, the spectra of N1, N2, N3, and N4 membranes exhibited the new absorption peaks at around1030 cm^−1^, which could be assigned to the C-O-C of ester. The new peaks suggest the occurrence of the interfacial polymerization between the -OH groups of β-CD and the acyl chloride groups of IPC.

### Membrane morphologies

**Figure 4:**
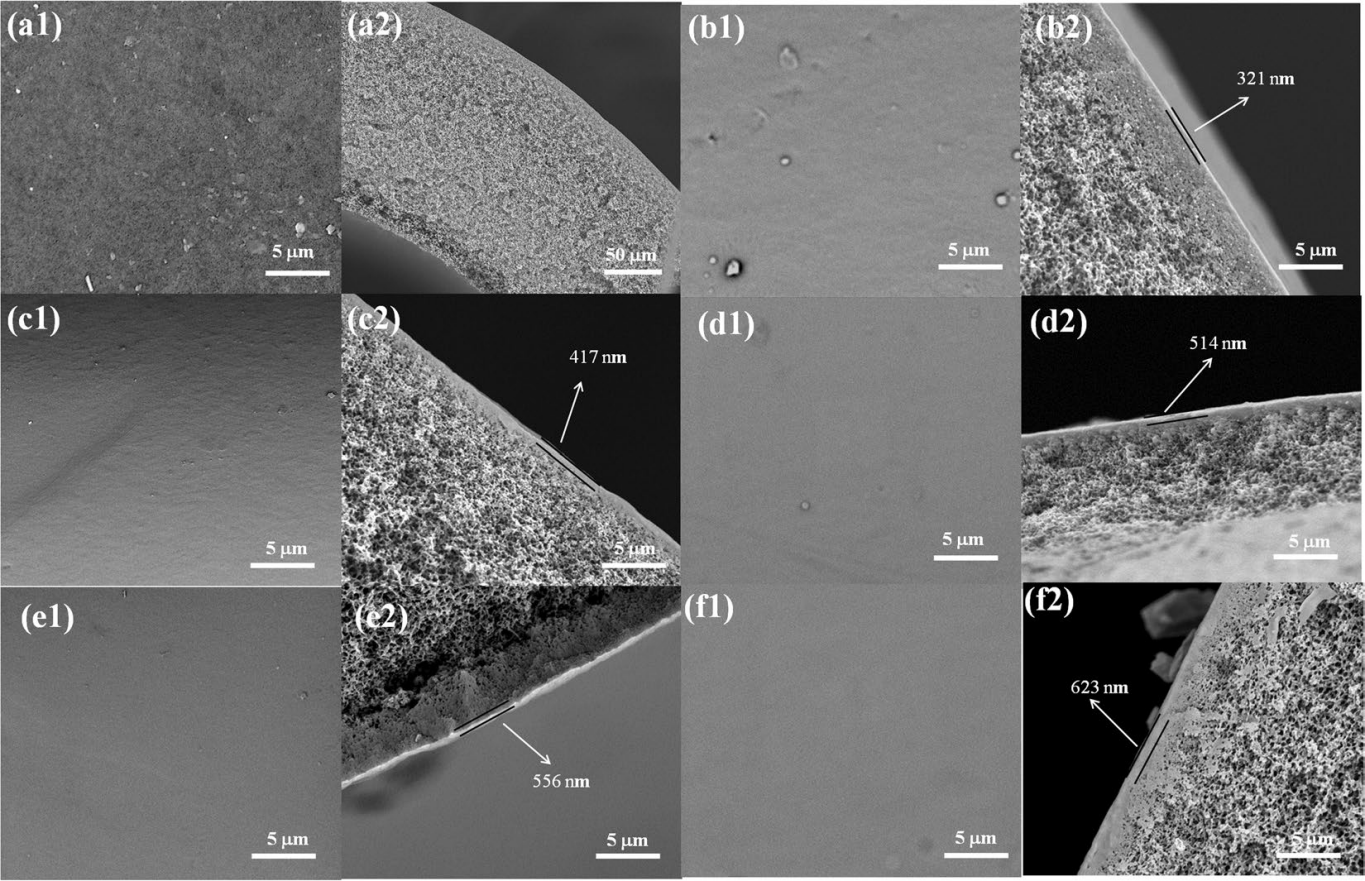
SEM images of (**a**) PVDF UF membrane, (**b**) NF-0, (**c**) NF-1, (**d**) NF-2, (**e**) NF-3 and (**f**) NF-4; (**a1–f1**: the surface, **a2–f2**: the cross section).

Figure 4 shows the surface and the cross-section morphologies of the PVDF UF and different resultant composite NF membranes. As shown in Fig. 4(a1), the surface of PVDF UF membrane presented a number of uniformly distributed small pores. Figure 4(b1–f1) showed the SEM images of different NF membrane surfaces. There was no perceptible pore observed in the inner surface. It could be seen clearly that the higher the β-CD concentration was, the smoother the resultant composite NF membrane surface was. Figure 4(a2–f2) showed the cross-section morphologies of the PVDF UF membranes and different hollow fiber composite NF membranes, from which a typical macrovoid structure of UF membrane could be observed. As seen from Fig. 4(b2–f2), there is a thin active layer attached to the surface of PVDF UF membrane. The thickness of the active layer increased gradually as the β-CD concentration increased from 0 to 2.0 wt.%. It demonstrated that a polyester film had formed and been bound to the polyamide layer tightly.

### Hydrophilicities of membrane surfaces

**Figure 5:**
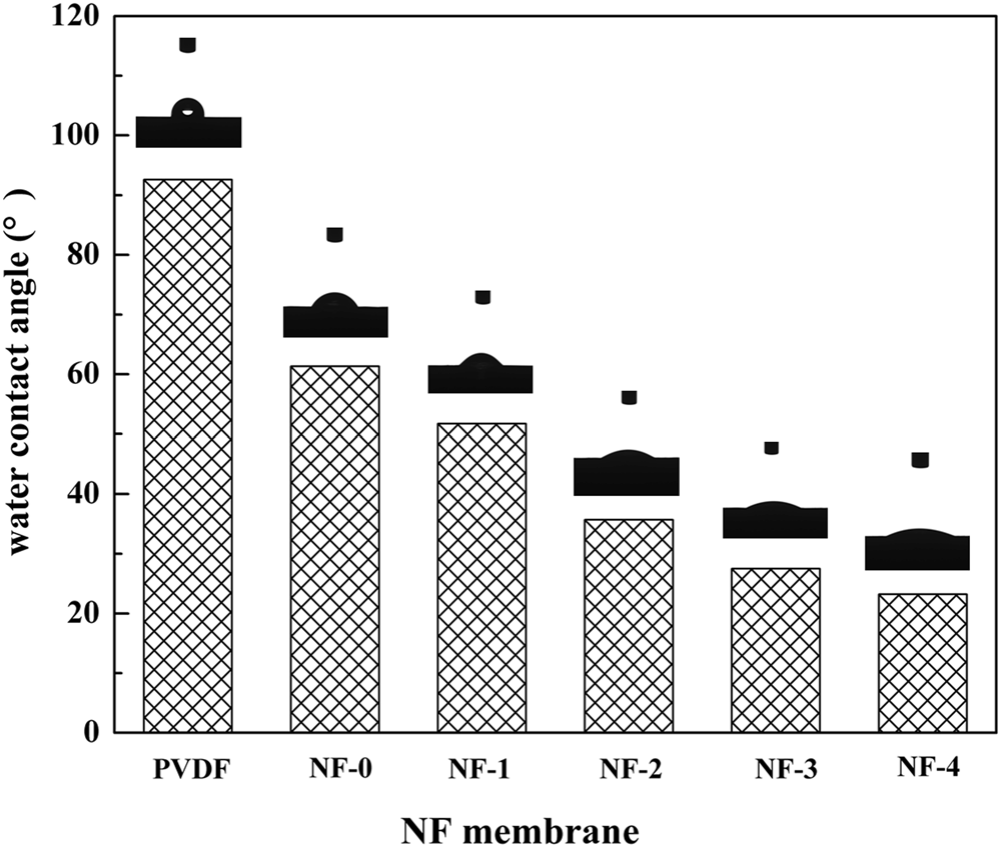
Static contact angles of PVDF UF membrane and different hollow fiber composite NF membranes.

Contact angle measurements were employed to characterize the hydrophilicities of the membrane surfaces. Figure 5 and Table 1 show the static contact angles of PVDF UF membrane and the resultant hollow fiber composite NF membranes. It could be seen clearly that the surface of PVDF UF membrane is hydrophobic with a WCA of 92.6°, and the resultant hollow fiber composite NF membranes are hydrophilic with relatively lower contact angles. The water contact angles decreased from 45° to 23° with increasing β-CD concentration from 0 to 2.0 wt.%. It suggested that the introduction of β-CD into the active layer effectively improve the hydrophilicity of the resultant composite NF membranes and play an important role in improving the resistance of the fouling^38,39^.

**Table 1:** The rejections and the permeate fluxes to different inorganic salts and water contact angles of the resultant hollow fiber composite NF membranes.

Membrane	WCA (°)	*R* (%)			*F* (L·m^−2^·h^−1^)		
		Na_2_SO_4_	MgSO_4_	MgCl_2_	Na_2_SO_4_	MgSO_4_	MgCl_2_
PVDF UF	92.6	—	—	—	—	—	—
NF-0	61.3	31.5	59.3	94.6	18.7	14.6	12.7
NF-1	51.7	32.4	60.3	94.3	20.6	17.9	13.8
NF-2	35.7	30.9	58.6	95.1	23.4	19.8	15.6
NF-3	27.5	34.9	61.2	94.7	27	22.4	19.8
NF-4	23.2	33.4	60.3	94.2	28.1	23.0.8	19.7

### Rejection performances

**Figure 6:**
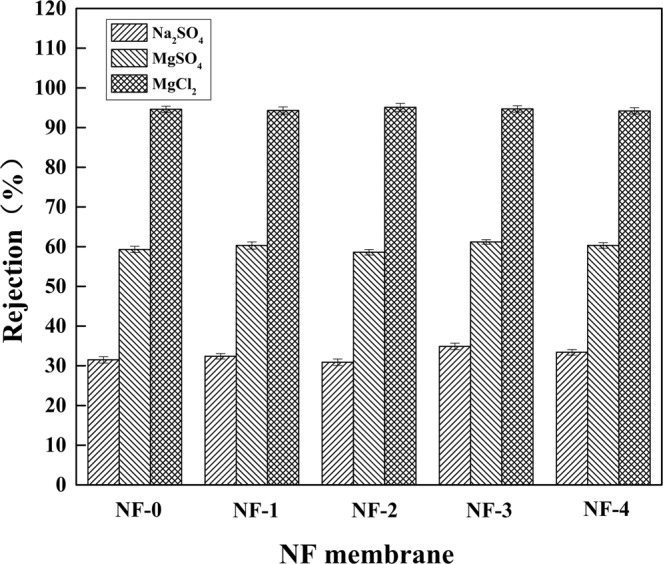
Effects of β-CD concentration on salt rejections of the hollow fiber composite NF membranes.

The influences of β-CD concentration on rejection performances were investigated. As shown in Table 1 and Fig. 6, the rejections were almost unaffected by β-CD concentration. In detail, the rejections (*R*) to 1000 ppm Na_2_SO_4_, MgSO_4_, and MgCl_2_ aqueous solutions were 33.4%, 60.2%, and 94.2%, respectively. According to the decreasing order of the rejections to inorganic salts, the resultant hollow fiber composite NF membranes were positively charged with stronger repulsion to di/multi-valence cations and stronger attraction to di/multi-valence anions, which could be attributed to Donnan exclusion theory^40^.

**Figure 7:**
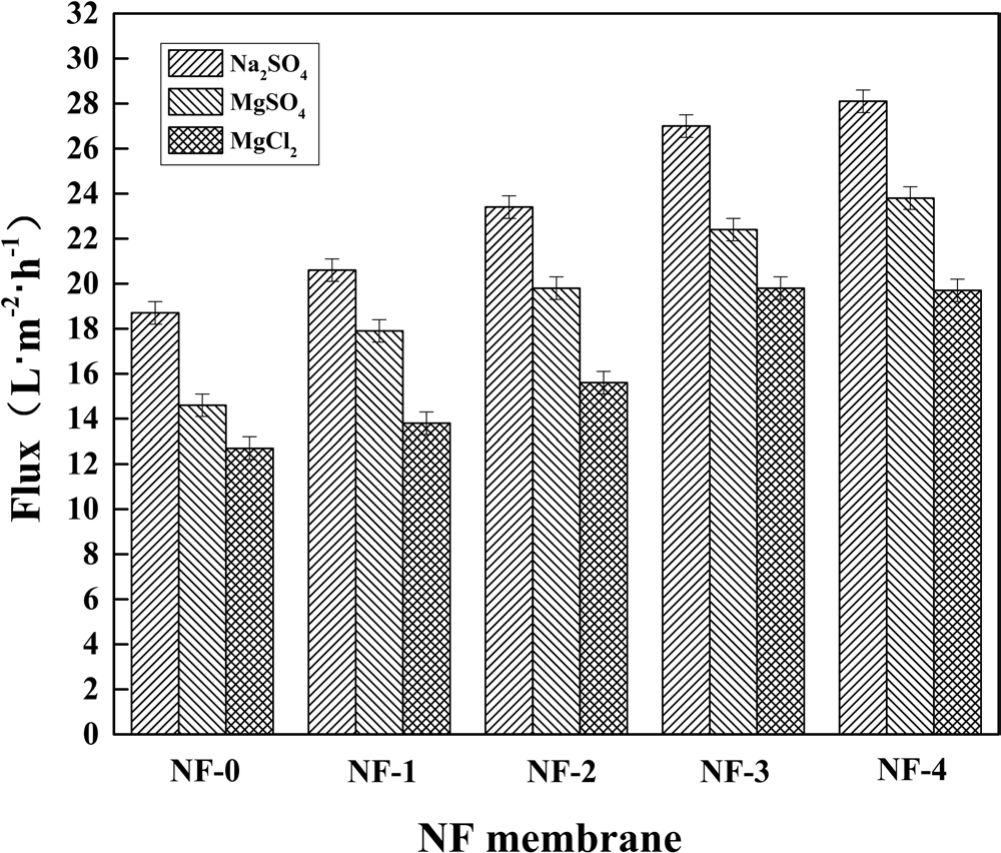
Effects of β-CD concentration on permeate fluxes of the hollow fiber composite NF membranes.

Figure 7 shows the permeate flux of various hollow fiber composite NF membranes. It could be seen clearly that as the feeds were Na_2_SO_4_, MgSO_4_, and MgCl_2_ aqueous solutions, respectively, all the permeate fluxes increased significantly with the increase of β-CD concentration. This could be explained with two reasons. Firstly, since β-CD contains a large amount of hydroxyl (-OH) groups, the introduction of β-CD into the active layer enhances the hydrophilicity of the composite NF membrane surface. The hydrogen bond interaction between water molecules and membrane surface was enhanced, facilitating water to transport through the composite membrane. Secondly, by introducing the β-CD molecules into the membranes, the special structure of β-CD could form the water channels, which would facilitate water to pass through more easily as well.

**Figure 8:**
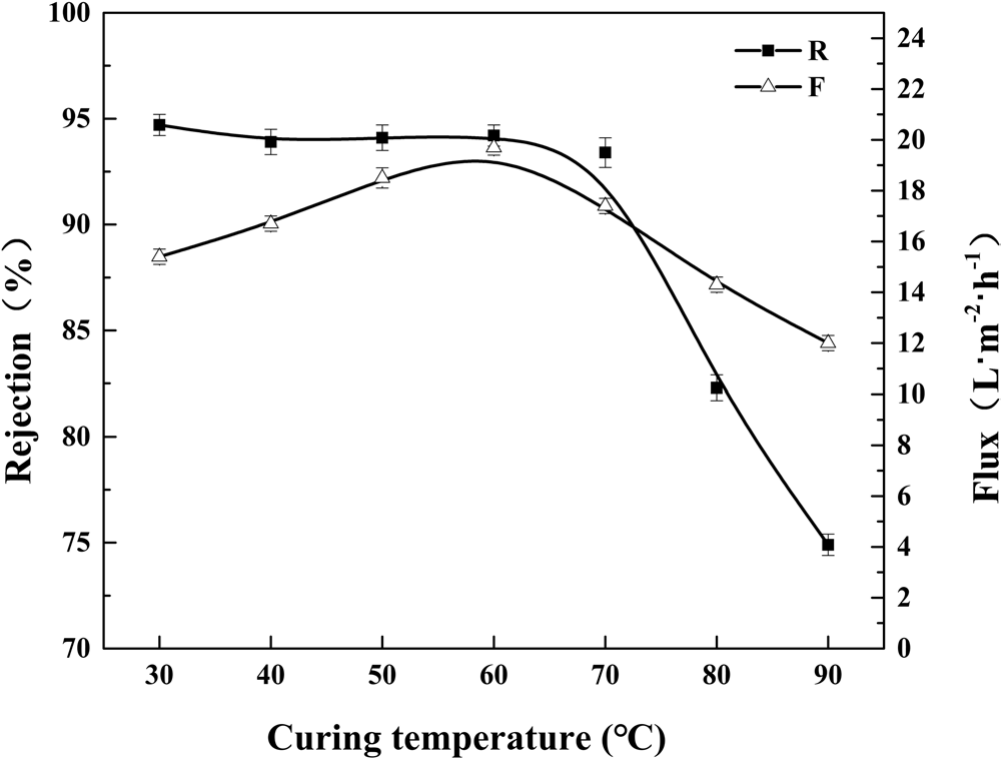
Effects of the post-heat curing temperature on the rejection and flux performances of the composite hollow fiber NF membranes.

The effects of different post-heat curing temperature in the range of 30 to 90 °C were investigated. The rejection performances were evaluated with the permeation tests using 1000 ppm MgCl_2_ aqueous solution at 25 °C and 0.2 MPa. As could be seen from Fig. 8, the rejection (*R*) to MgCl_2_ aqueous solution changed little with increasing the curing temperature in the range of 30 to 70 °C, and then decreased as the curing temperature was higher than 70 °C. The permeate flux increased gradually as the curing temperature increased from 30 to 60 °C, and then showed a decline as the curing temperature continued to rise. This might be because the higher curing temperature could accelerate the reaction between β-CD and acyl chloride, and also increase the cross-linking degree of the polymer nets, resulting in a denser active layer, thus the permeate flux decreased as the curing temperature was above 60 °C. On the other hand, the base membrane shrunk further at a relatively higher curing temperature, the active layer was not bound to the base membrane tightly, resulting in a decrease in the rejection (*R*).

### Anti-fouling performance of the hollow fiber composite NF membranes

**Figure 9:**
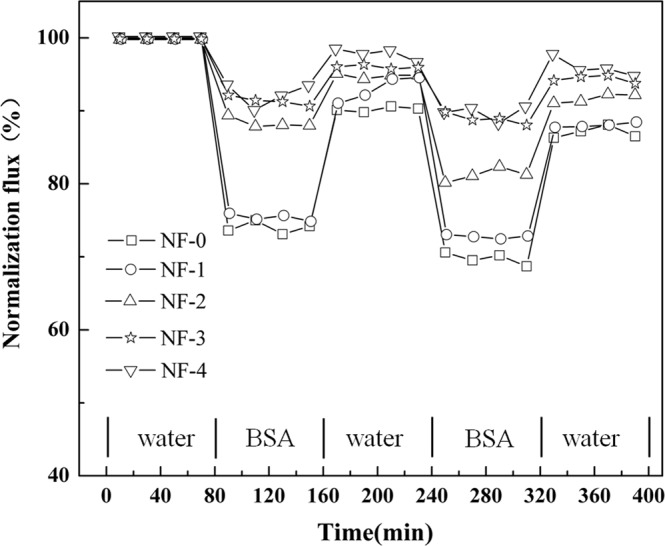
Time-dependent recovery rate for permeate flux tested with DI water and BSA/PBS aqueous solution.

The anti-fouling performance was investigated by permeation test using DI water and BSA/phosphate-buffered saline solution (pH: 7.4) as the model feed solution at 25 °C and 0.2 MPa. It could be observed from Fig. 9 that the reduced rate of permeate flux decreased gradually with the increase in β-CD concentration. After 80-min running with the BSA solution, the permeate fluxes of NF-0, NF-1, NF-2, NF-3, and NF-4, maintained at 74.2%, 75.8%, 88.2%, 90.8%, and 93.3%, respectively. Then the NF membranes were cleaned with DI water for 30 min, the recovery rates of the five NF membranes for the permeate fluxes were 89.7%, 93.2%, 95.0%, 96.2%, and 97.6%, respectively. The NF-4 shows the best anti-fouling performance with normalized flux of 93.3%. It could be explained by the reason that the hydrophilicities of the resultant membrane surfaces were enhanced with the abundant –OH groups in β-CD. The membrane surfaces’ binding to water were enhanced, while the protein absorption on it was reduced^41^.

**Figure 10:**
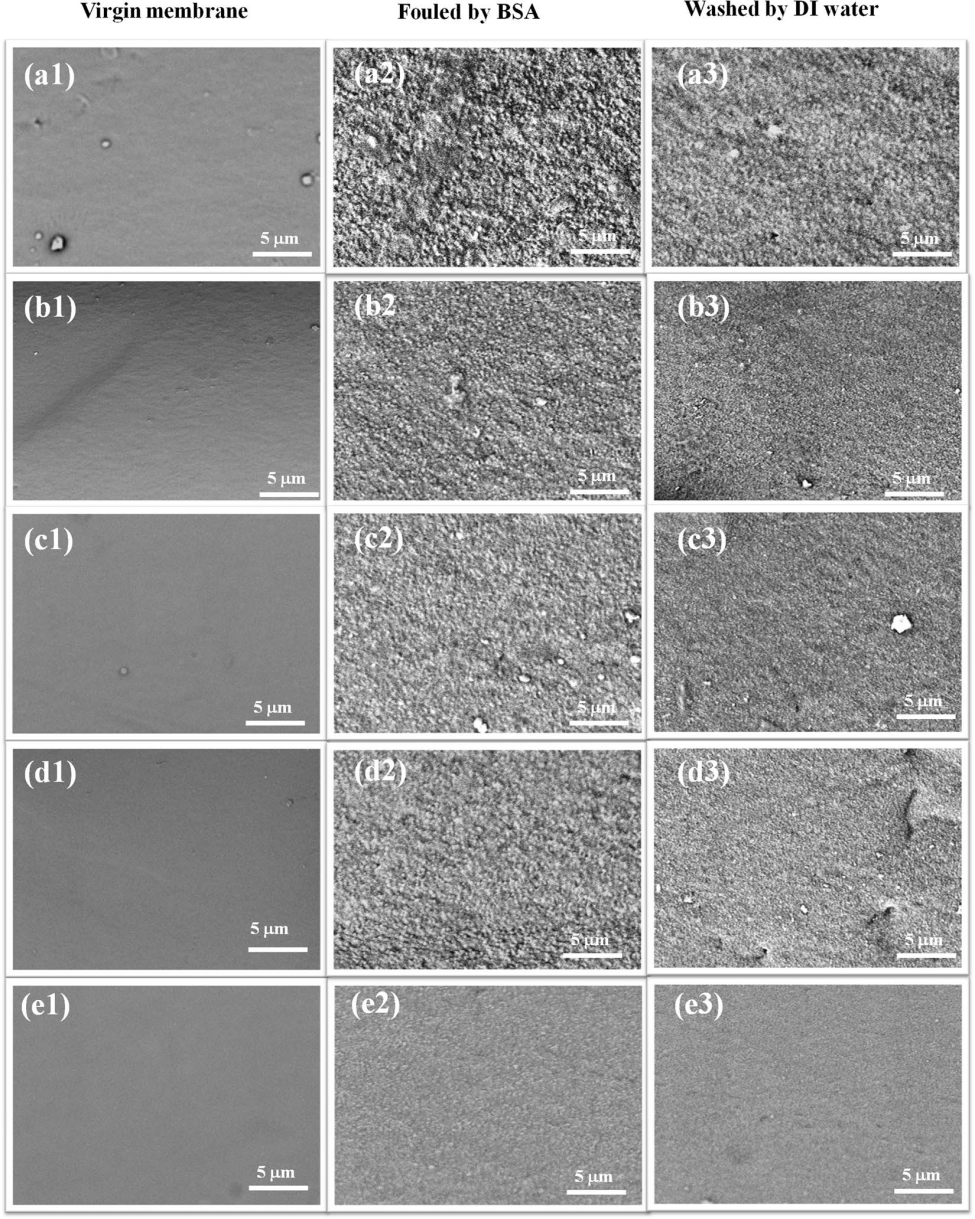
SEM surface images of the virgin membranes, fouled membranes and refreshed membranes: (**a1–a3**) NF-0, (**b1–b3**) NF-1, (**c1–c3**)NF-2, (**d1–d3**) NF-3, (**e1–e3**) NF-4.

In order to further identify its anti-fouling properties, the surface morphologies of the original membranes, the fouled membranes, and the refreshed membranes with different β-CD concentration were characterized with SEM. As shown in Fig. 10, with the increase in β-CD concentration, the NF membrane surface exhibited less protein absorption. Moreover, after washed with DI water, it is easier for the NF membranes that fabricated with high β-CD concentration to remove the absorbed BSA from the surface. It might be because that the hollow fiber composite NF membranes containing more β-CD have smoother surfaces and more shallow grooves, which could make it easier to wash the BSA away from the membrane surface.

## Conclusions

Anti-fouling hollow fiber composite NF membranes were prepared via interfacial polymerization and layer-by-layer method using polyethylenimine (PEI), isophthaloyl dichloride (IPC), and β-cyclodextrin (β-CD) as the monomer of the aqueous phase, the monomer of the organic phase, and the surface modification agent, respectively. The resultant hollow fiber composite NF membranes showed their positively charged characteristics and high rejections to inorganic salts. The introduction of β-CD into the active layer of the hollow fiber composite NF membranes improved the surface hydrophilicity of the resultant composite membranes and the permeate flux significantly. The permeate flux for 1000 ppm MgCl_2_ aqueous solution increased from 12.7 L·m^−2^·h^−1^ to 19.7 L·m^−2^·h^−1^ at 0.2 MPa and room temperature as increasing the β-CD concentration from 0 to 2.0 wt.%. The resultant hollow fiber composite NF membranes showed excellent anti-fouling property. After 80-min running with BSA solution, the recovery rate for permeate flux was 93.3%. Furthermore, the recovery rate could be increased up to 97.6% after being washed with DI water.
